# Can pegylated interferon improve the outcome of polycythemia vera patients?

**DOI:** 10.1186/s13045-017-0395-1

**Published:** 2017-01-13

**Authors:** Elena Crisà, Marco Cerrano, Eloise Beggiato, Giulia Benevolo, Giuseppe Lanzarone, Paola Maria Manzini, Alessandra Borchiellini, Ludovica Riera, Mario Boccadoro, Dario Ferrero

**Affiliations:** 1 Hematology Division, Università degli Studi di Torino, Via Genova 3, 10126 Turin, Italy; 2 S.C. Hematology, A.O. Città della Salute e della Scienza, Turin, Italy; 3 Transfusion Medicine Unit, A.O. Città della Salute e della Scienza, Turin, Italy; 4 Section of Pathology, Department of Molecular Biotechnology and Health Sciences, University of Torino, Turin, Italy

**Keywords:** Pegylated interferon, Polycythemia vera, JAK2 allele burden, Hydroxyurea

## Abstract

Pegylated interferon (peg-IFN) was proven by phase II trials to be effective in polycythemia vera (PV); however, it is not clear whether it could improve patient outcome compared to hydroxyurea (HU). Here, we present an observational study on 65 PV patients aged 65 years or younger, who received either peg-IFN (30) or HU (35) according to the physician choice. Median follow-up was 75 months. The two cohorts were comparable for patient and disease characteristics. Eighty-seven percent of the patients treated with peg-INF responded, with a CR rate of 70% as compared to 100 and 49% with HU, respectively. Discontinuation rate was similar in the two groups (20% in peg-IFN vs 17% in HU). JAK2 allele burden was monitored in peg-INF arm only, and a reduction was observed in 88% of the patients. No thrombotic events were observed during peg-IFN treatment compared to three on HU. Disease progression to myelofibrosis or acute myeloid leukemia occurred to a patient only in peg-INF, compared to three in HU. Overall, three second malignancies were observed during the study, two in patients who received HU only, and one in a patient largely treated HU who received also peg-IFN for 3 months. Overall survival was significantly better for peg-IFN patients compared to HU, *p* = 0.027. Our study, albeit limited by small patient and event number and lack of randomization, confirms the efficacy of peg-INF in PV and shows a significant survival advantage for peg-INF-treated patients. Waiting for confirming data from the ongoing phase III trials, our study can support peg-INF as a first-line treatment option for PV, at least for younger patients.

## Main text

The outcome of patients with polycythemia vera (PV) is mainly affected by thromboembolic events and evolution to myelofibrosis (MF) and acute myeloid leukemia (AML). Currently approved treatments for PV reduce thrombosis incidence but do not seem to have any impact on the risk of disease progression [1]. Recently, pegylated interferon (peg-IFN) was found to be effective in PV, with hematologic response rate up to 100%, complete response (CR) rate ranging from 54% to more than 90% and very low thrombosis incidence [2, 3]. Moreover, the majority of patients also experienced a reduction of JAK2 allele burden, thus suggesting that peg-INF might be able to modify the natural history of PV [4]. However, the studies published so far did not address the question of whether peg-INF could reduce disease progression rate and eventually improve long-term survival compared to hydroxyurea (HU), the actual gold standard treatment in PV [1]. Here, we report our observational study comparing peg-INF with HU in a population of PV patients below 65 years.

Since 2010, peg-INF becomes available off-label in our Institution. Patients diagnosed with PV according to WHO 2008 classification, aged 65 years or younger, with normal cardiac, renal, and liver function, and without history of autoimmune disease were eligible for this study. According to the physician choice, newly diagnosed patients requiring cytoreductive treatment could receive either peg-INFalpha-2a (Pegasys, Roche) or HU and patients previously treated with HU could be switched to peg-INF regardless of the response achieved. Data were prospectively collected in the Registry of Myeloproliferative Neoplasms of our Institution and the study received Ethic Committee approval. All patients signed an informed consent in accordance with the Declaration of Helsinki. JAK2 quantitative level was monitored every 6 months in the peg-IFN arm only. CR was defined according to European LeukemiaNet 2009 criteria [5]. Treatment with peg-INF was started subcutaneously at 90 μg weekly and increased to 135 μg weekly if tolerated. The dose was decreased in case of intolerance or cytopenia. HU dose could range from 500 up to 2000 mg orally per day and was modulated according to hematological response.

Two different populations were compared: patients who received peg-IFN vs the control group receiving HU only. Patients’ characteristics were compared using Fisher’s exact test for the categorical variables and the Kruskal-Wallis test for the continuous ones. Overall survival was estimated from the study start until death or last follow-up by Kaplan-Meier method; any statistical difference between curves was assessed by log-rank test.

Sixty-five patients were included, with a median follow-up of 75 months (range 14–80 months). Thirty patients were treated with peg-INF and 35 with HU only. The two cohorts were comparable for gender, age at diagnosis, age at treatment start, time from diagnosis to study entry, previous thrombotic events and cardiovascular risk factors (see Table 1). In the peg-INF group, 19 patients (63%) had previously received HU for a median time of 50 months (range 2–120 months) and were in hematological response. There was not any significant difference in terms of age, gender, and disease characteristics between patients treated front line with peg-INF and those who previously received HU.

**Table 1:** Patients characteristics

	peg-INF group	HU group	*p* value
Sex, *n* (%)			
Male	19 (63)	23 (66)	0.841
Female	11 (37)	12 (34)	
Age at diagnosis (years)			
Median (range)	49 (18–64)	53 (29–65)	0.180
Age at treatment start			
Median (range)	54 (32–65)	55 (36–65)	0.645
Time from diagnosis to study entry (months)			
Median (range)	11 (0–179)	18 (0–169)	0.631
Palpable spleen			
*N* (%)	16 (55)	14 (42)	0.316
Cardiovascular risk factors			
*N* (%)	16 (53)	20 (57)	0.758
Previous thrombosis			
*N* (%)	5 (17)	9 (26)	0.376

Globally, 87% of the patients treated with peg-INF responded (26/30), with a CR rate of 70% (21/30). Median time to CR was 6 months and median peg-INF dose at CR was 90 μg weekly. The four patients who did not respond had to early discontinue treatment due to intolerance. Most patients (87%) experienced some adverse events: hematologic toxicity (43%, grade 3–4 7%), flu-like symptoms (30%), and liver test elevation (23%, grade 3–4 7%). Overall discontinuation rate was 20%. JAK2 allele burden reduction was observed in 88% of the patients (21/24 evaluable ones). Median JAK2 allele burden at diagnosis was 40.5% (range 1.5–91%), and it decreased to 17% (range 0.3–81%) and 15.8% (range 0–77%) at 12 and 24 months after treatment start, respectively.

**Fig. 1:**
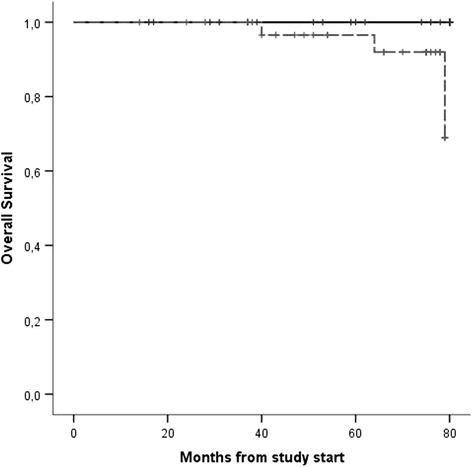
Overall survival from study start by intention to treat principle. peg-INF group (*continuous line*) vs HU group (*dashed line*)

All patients in the HU group responded, with a CR rate of 49%, and discontinuation rate was 17%. In the peg-INF group, only one patient, who already had grade 1 bone marrow fibrosis, progressed to overt post-PV MF compared to three in the HU group, of whom two subsequently transformed to AML. Three second malignancies were observed overall, two in the HU group, and one in a patient treated with HU for 48 months and with peg-INF for 3 months only. No thrombotic events were observed during peg-IFN treatment compared to three on HU. The landmark analysis from study start showed a significantly better survival for peg-IFN patients, *p* = 0.027 (Fig. 1), with no death observed compared to three in the HU group. No other variables such as sex, age, cardiovascular risk factors, and previous thrombosis had a significant impact on survival.

HU is widely considered the first-line cytoreductive therapy for PV [1]. As a matter of fact, data supporting peg-INF use come from phase II studies only, while phase III trials comparing peg-INF to HU are ongoing. However, these data will be completely available only in several years. Therefore, some experts are now considering the possibility of using peg-INF front-line in younger PV patients given its ability to reduce JAK2 allele burden, which could impact on the natural history of the disease and its long-term safety [6]. Our experience confirms the efficacy and tolerability of peg-INF, with few grade 3 or 4 adverse events and a discontinuation rate comparable to that of HU. Moreover, no thrombosis was observed during peg-INF treatment, suggesting the non-inferiority of this drug to HU in preventing thrombotic events even in patients at high thrombotic risk. Furthermore, we observed no AML progression, and one case only of evolution to MF in the peg-INF group, together with a reduction in JAK2 allele burden in the majority of patients. While alkylating agents have been shown to increase AML progression risk, HU has never been proven to be leukemogenic [1]. However, concerns about long-term exposure to this drug in young patients do exist [6]. Besides, a recent report showed an increased incidence of second malignancies in patients treated with HU compared to INF [7].

Our study, which has a substantial follow-up duration but which is limited by small patient and event number and lack of randomization, shows a significant survival advantage for peg-INF-treated patients, a data that could be explained by the absence of thrombotic events, the modest but important reduction in MF and AML progression and, possibly, the decreased risk of second malignancies in the peg-INF group. Indeed, our data require confirmation, but until the ongoing phase III trials give conclusive answers, they could support peg-INF as a first-line treatment option for PV, at least for younger patients.

## Abbreviations

AML: Acute myeloid leukemia
CR: Complete response
HU: Hydroxyurea
MF: Myelofibrosis
peg-INF: Pegylated interferon
PV: Polycythemia vera
